# A Model for Carbohydrate Metabolism in the Diatom *Phaeodactylum tricornutum* Deduced from Comparative Whole Genome Analysis

**DOI:** 10.1371/journal.pone.0001426

**Published:** 2008-01-09

**Authors:** Peter G. Kroth, Anthony Chiovitti, Ansgar Gruber, Veronique Martin-Jezequel, Thomas Mock, Micaela Schnitzler Parker, Michele S. Stanley, Aaron Kaplan, Lise Caron, Till Weber, Uma Maheswari, E. Virginia Armbrust, Chris Bowler

**Affiliations:** 1 Fachbereich Biologie, University of Konstanz, Konstanz, Germany; 2 School of Botany, University of Melbourne, Melbourne, Victoria, Australia; 3 EA 2663, Faculty of Science, University of Nantes, Nantes, France; 4 School of Oceanography, University of Washington, Seattle, Washington, United States of America; 5 Scottish Association of Marine Science, Dunstaffnage Marine Laboratory, Oban, Argyll, United Kingdom; 6 Department of Plants and Environmental Sciences, The Hebrew University of Jerusalem, Jerusalem, Israel; 7 U533, INSERM, Faculty of Medecine, University of Nantes, France; 8 Centre National de la Recherche Scientifique (CNRS), UMR8186, Ecole Normale Supérieure, Paris, France; 9 Cell Signalling Laboratory, Stazione Zoologica, Villa Comunale, Naples, Italy; Max Planck Institute for Chemical Ecology, Germany

## Abstract

**Background:**

Diatoms are unicellular algae responsible for approximately 20% of global carbon fixation. Their evolution by secondary endocytobiosis resulted in a complex cellular structure and metabolism compared to algae with primary plastids.

**Methodology/Principal Findings:**

The whole genome sequence of the diatom *Phaeodactylum tricornutum* has recently been completed. We identified and annotated genes for enzymes involved in carbohydrate pathways based on extensive EST support and comparison to the whole genome sequence of a second diatom, *Thalassiosira pseudonana*. Protein localization to mitochondria was predicted based on identified similarities to mitochondrial localization motifs in other eukaryotes, whereas protein localization to plastids was based on the presence of signal peptide motifs in combination with plastid localization motifs previously shown to be required in diatoms. We identified genes potentially involved in a C4-like photosynthesis in *P. tricornutum* and, on the basis of sequence-based putative localization of relevant proteins, discuss possible differences in carbon concentrating mechanisms and CO_2_ fixation between the two diatoms. We also identified genes encoding enzymes involved in photorespiration with one interesting exception: glycerate kinase was not found in either *P. tricornutum* or *T. pseudonana*. Various Calvin cycle enzymes were found in up to five different isoforms, distributed between plastids, mitochondria and the cytosol. Diatoms store energy either as lipids or as chrysolaminaran (a β-1,3-glucan) outside of the plastids. We identified various β-glucanases and large membrane-bound glucan synthases. Interestingly most of the glucanases appear to contain C-terminal anchor domains that may attach the enzymes to membranes.

**Conclusions/Significance:**

Here we present a detailed synthesis of carbohydrate metabolism in diatoms based on the genome sequences of *Thalassiosira pseudonana* and *Phaeodactylum tricornutum.* This model provides novel insights into acquisition of dissolved inorganic carbon and primary metabolic pathways of carbon in two different diatoms, which is of significance for an improved understanding of global carbon cycles.

## Introduction

Diatoms are abundant unicellular algae in aquatic habitats. They can produce enormous amounts of biomass and are thought to be responsible for about 20% of global carbon fixation. As much as 16 gigatons of the organic carbon produced by marine phytoplankton per year, or about one third of total ocean production is thought to sink into the ocean interior preventing re-entrance of this carbon into the atmosphere for centuries [1]. Recent assessments suggest that diatom-mediated export production can influence climate change through uptake and sequestration of atmospheric CO_2_ [2], [3]. The role diatoms play in mitigating atmospheric CO_2_ concentrations is of special interest now with the rising levels of this “greenhouse gas” and consequent global warming. A significant fraction of the organic carbon generated by diatoms remains in the upper ocean and supports production by higher trophic levels and bacteria.

Despite the important role of diatoms in aquatic ecosystems and the global carbon cycle, relatively little is known about carbon fixation and carbohydrate pathways in these algae [4]. For example the exact mode of CO_2_ fixation is largely unsolved. Ribulose-1,5-bisphosphate carboxylase/oxygenases (Rubisco) from diatoms have half-saturation constants for CO2 of 30–60 µM [5] despite the fact that typical sea water contains about 10 µM CO_2_ [6]. To prevent potential CO_2_ limitation, most diatoms have developed mechanisms to concentrate dissolved inorganic carbon (DIC) via a CO2 concentrating mechanism (CCM) [7]. Although most of the Calvin cycle enzymes in diatoms are very similar to those in land plants, there are indications that they may be differently regulated by light [8]. Furthermore, some metabolic pathways appear to be missing altogether from diatoms [9]. Finally, there is only scarce information available on the localization, synthesis and storage of chrysolaminaran, the principle storage carbohydrate in diatoms. Diatoms may produce and secrete vast amounts of carbohydrates that play important roles in phototrophic biofilms, yet very little is known about synthesis and secretion of these carbohydrates.

Research on diatoms advanced significantly with publication of the whole genome sequences of the centric diatom *Thalassiosira pseudonana* [10] and of expressed sequence tags (ESTs) from the pennate diatom *Phaeodactylum tricornutum* [11]. Recent availability of whole genome sequence and about 100,000 ESTs for *Phaeodactylum tricornutum* provide additional opportunities to understand unique physiological characteristics of diatoms. Together with new experimental resources such as genetic transformation, now feasible for several diatom species [12]–[14] and various laboratory-based studies of their physiology [8], diatoms have become model photosynthetic representatives for non-green algae.

Diatoms have an evolutionary history distinct from higher plants. Diatoms are eukaryotic chimeras derived from a non-photosynthetic eukaryote that domesticated a photoautotrophic eukaryotic cell phylogenetically close to a red alga [15]. After incorporation, the endosymbiont was successfully transformed into a plastid that retained a small plastid genome, but lost the nuclear and the mitochondrial genomes as distinct entities. In addition to the genetic consequences that resulted from extensive gene transfer events and genomic reorganization, secondary endocytobiosis also increased the complexity of diatom cell structure, with implications on physiology and biochemistry. A significant difference between diatom plastids and those of higher plants is that diatom plastids are surrounded by four rather than two membranes, the outermost of which is contiguous with the endoplasmic reticulum. This means that import of all nuclear-encoded plastid proteins and the exchange of metabolites like carbohydrates between the plastids and the cytoplasm must take place across four membranes. To accomplish this task, nuclear-encoded proteins imported into diatom plastids possess an N-terminal signal peptide that targets the protein first to the endoplasmic reticulum and a plastid localization peptide that targets the protein to plastid stroma [16], [17]. Another striking difference between diatoms and green algae/land plants is their different nuclear and mitochondrial backgrounds because they arose from different host cells.

We annotated genes involved in carbon acquisition and metabolism in the genome of the diatom *P. tricornutum* and compared these gene models to the only other diatom whole genome sequence of *Thalassiosira pseudonana*. The 5′-most ends of a majority of critical genes were identified based on EST support. This meant that N-terminal leader sequences could be predicted for most proteins and thus their targeting to different compartments within the cell. Here, we present a comprehensive model of the localization of enzymes and pathways involved in carbon assimilation and carbohydrate production and catabolism.

## Results and Discussion

### Structure of the genome and gene annotation

Following publication of the draft *Thalassiosira pseudonana* Hasle & Heimdal (CCMP 1335) genome [10], a majority of sequence gaps were closed at the Stanford Human Genome Center (SHGC; Stanford, CA, USA) and a new version of the genome sequence is now publicly available at http://genome.jgi-psf.org/Thaps3/Thaps3.home.html. A second diatom genome, from *Phaeodactylum tricornutum* Bohlin (CCAP1055/1), was subsequently sequenced and completed at the U.S. Department of Energy Joint Genome Institute (JGI, http://www.jgi.doe.gov/, Walnut Creek, CA, USA) and SHGC, and is available publicly at http://genome.jgi-psf.org/Phatr2/Phatr2.home.html. In addition, 100,000 ESTs generated from *P. tricornutum* cells grown in 14 different conditions have been generated by Genoscope (Evry, France) and are available at http://www.biologie.ens.fr/diatomics/EST. Both genomes are approximately 30 Mb and contain between 10,000 and 11,500 genes. Assembly and annotation of the whole genome of *P. tricornutum* will be published separately (manuscript in preparation). Here we focus solely on those pathways involved in carbohydrate metabolism. In the following sections, we include protein IDs (Prot-ID) from version 2.0 (*P. tricornutum*) of the JGI sequence database in parentheses. See Table S1 for a list of annotated genes together with the Prot-IDs in *T. pseudonana*.

### Prediction of intracellular targeting

Nuclear encoded proteins are translated in the cytosol and subsequently transported to their respective target locations. In most known cases an N-terminal targeting domain can send the proteins into the ER, mitochondria, plastids, the extracellular space or to other compartments. In land plants relatively similar transit peptides are used to target into plastids or mitochondria, making it sometimes difficult to predict the correct compartment. Mitochondrial import sequences in diatoms are similar to those in other eukaryotes. Diatom plastid presequences, however, differ significantly from those of land plants or green algae [16]. Diatom plastids are surrounded by four membranes, the outermost being studded with ribosomes and continuous with the endoplasmic reticulum (ER) [18]. Nuclear encoded plastid proteins of diatoms contain N-terminal bipartite presequences consisting of a signal peptide followed by a transit peptide-like domain. Such presequences are easily recognized due to an essential targeting motif with a characteristic signature at the signal peptide cleavage site [16], [17].

### CO_2_ fixation: a biochemical (C4) or a biophysical CCM-like metabolism?

The apparent photosynthetic affinity of diatoms for inorganic carbon (C_i_) is considerably higher than expected based on the affinity of their Rubisco for CO_2_ [5]. Extensive diatom blooms that occur during large iron fertilization experiments in high nutrient low chlorophyll regions of the oceans [19], [20] suggest that diatoms are not CO_2_ limited under natural oceanic conditions. Both results imply that diatoms possess efficient CO_2_ concentrating mechanisms (CCM), although underlying mechanisms (either a biochemical C4 or a biophysical CCM, or both) are still controversial (see [3], [4]).

Studies on the biochemistry of photosynthesis in the well-characterized marine diatom *Thalassiosira weissflogii* suggested that a C4-like pathway could exist whereby a C4 compound such as malate or OAA is decarboxylated, typically within the chloroplast, to deliver CO_2_ to Rubisco [21], [22]. The possibility of a C4-like pathway in the related species *T. pseudonana* was examined based on an *in silico* analysis of gene content [10]. The *T. pseudonana* genome appears to encode the enzymes phosphoenolpyruvate carboxylase (PEPC), phospoenolpyruvate carboxykinase (PEPCK) and pyruvate orthophosphate dikinase (PPDK). Each of these enzymes is required for C4-metabolism, although they also play a role in C3-metabolism. Subsequent analysis of transcript abundances for the putative C4-related genes in *T. pseudonana* indicated that the gene encoding PEPCK was up-regulated about 1.5 fold under reduced CO_2_ concentrations, whereas expression of genes encoding PEPC and PPDK were unaffected [3]. Despite the presence of typical C4 enzymes in both *Thalassiosira* species, short ^14^CO_2_ labelling experiments showed marked differences between them [4]. In *T. weissflogii*, about 30% of the ^14^C label (in 5 sec. experiments) was observed in malate and about 40% in triose phosphates. In contrast, in *T. pseudonana* production of ^14^C-labeled C4 products was negligible. Roberts et al. [4] concluded that a typical C3 metabolism occurs in *T. pseudonana*, despite the presence of C4 enzymes, whereas an intermediate C3-C4 may function in *T. weissflogii*.

**Figure 1:**
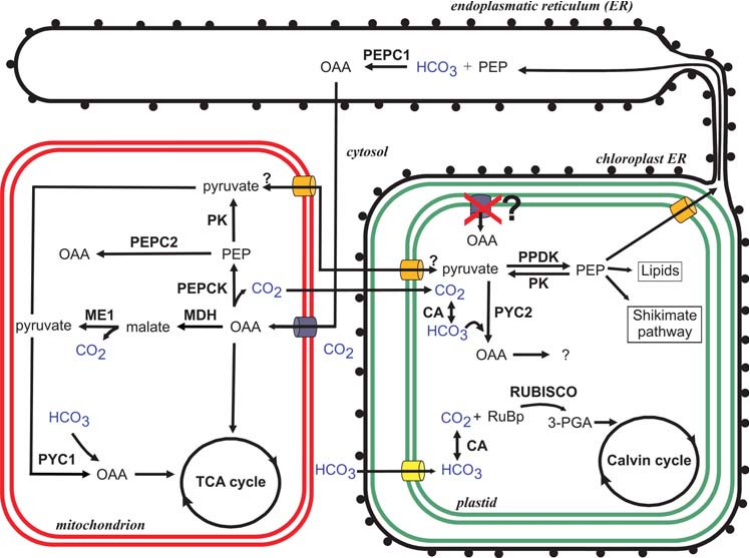
Model of carbon concentrating mechanisms (CCM) in diatoms based on annotations of the *Phaeodactylum tricornutum* and *Thalassiosira pseudonana* genomes. For discussion of the pathways see text. Enzyme abbreviations: CA: carbonic anhydrase; MDH: malate dehydrogenase; ME1: NAD malic enzyme, mitochondrial; PEPC: phosphoenolpyruvate carboxylase; PEPCK: phosphenolpyruvate carboxykinase; PK: pyruvate kinase; PPDK: pyruvate-phosphate dikinase; PYC: pyruvate carboxylase; RUBISCO: ribulose-1,5-bisphosphate carboxylase.

Genes essential for C4 metabolism were identified in *P. tricornutum*. A PPDK (21988), which catalyzes the formation of PEP, was identified and includes both a signal peptide and a putative plastid targeting sequence suggesting that PEP is generated in the plastid (Fig. 1). Two genes encoding PEPC have been identified (Fig. 1). The predicted protein sequence for one of them (PEPC1, 56026) has a high degree of identity (ca. 40% amino acid identity) with the PEPCs from green algae and higher plants. It possesses a signal peptide, but a plastidic transit peptide was not detected suggesting that this protein is targeted either to the ER or to the periplastidic space of the plastids [17]. A second PEPC (PEPC2, 20853) has high similarity (ca. 40% amino acid identity) to PEPCs from bacteria and contains a predicted mitochondrial targeting presequence. Decarboxylation of OAA appears to occur via a mitochondrial-localized PEPCK (23074). This enzyme has the greatest similarity to PEPCK from the proteobacterium *Campylobacter jejuni* (58% amino acid identity). Two additional decarboxylating enzymes belonging to the malic enzyme family were identified (27477, 56501) and apparently both possess a mitochondrial presequence. One of these enzymes (ME1) (56501) is characterized by a dinucleotide binding site that binds NAD rather than NADP. Thus, *P. tricornutum* and *T. pseudonana* appear to have two mitochondrial malic enzymes that are either NAD- or NADP-dependent. Genes encoding a mitochondrial-localized malate dehydrogenase (MDH) (51297), a pyruvate-kinase (PK6) (56172) and a pyruvate-carboxylase (PYC1) (30519) were also identified (Fig. 1). The respective substrates for this pathway may be transported into the mitochondria by a putative mitochondrial oxoglutarate/malate transporter (8990).

Models were developed to evaluate how such a C4-like carbon fixation pathway could operate in *P. tricornutum* (see our working scheme, Fig. 1). The first hypothesized step in carbon fixation is delivery of HCO_3_^−^ into cells either via specific transporters or by diffusion of CO_2_ and its subsequent conversion to HCO_3_^−^ through CA activity (see below). The hypothesized localization of PEPC1 (56026) to the ER or to that part of the ER that is connected to the plastid (CER) or to the periplastidic space (PPS) suggests that subsequent fixation of HCO_3_^−^ into a C4 compound likely occurs within either the ER or the periplastidic space. The localization of the C4 decarboxylation that delivers CO_2_ to Rubisco for fixation is not clear. Immuno-localization based studies provided early evidence that the decarboxylating enzyme PEPCK is located in the plastids of the centric diatom *Skeletonema costatum* [23]. Later, Reinfelder et al. [21] found that PEPCK activity co-localized with Rubisco activity in isolated plastid-enriched fractions from *T. weissflogii* and concluded that decarboxylation occurred within the plastids. Subsequent *in silico* analysis of both *P. tricornutum* and *T. pseudonana* indicated that the decarboxylating enzymes PEPCK and malic enzyme do not possess plastid targeting sequences. Moreover, there is no evidence that malate and/or oxaloacetate transporters in these organisms are localized to plastid membranes. Finally, addition of oxaloacetate to intact plastids isolated from the diatom *Odontella sinensis* [24] does not result in net O_2_ evolution as would be expected from the malic dehydrogenase reaction due to turnover of NADPH. Combined, these results suggest that subsequent decarboxylation steps required to generate CO_2_ for Rubisco delivery, at least in *P. tricornutum* and *T. pseudonana*, do not occur in the plastid.

Recent evidence with single chlorenchyma cells of the higher plants *Bienertia cycloptera* and *Borszczowia aralocaspica* provides support for a compartmentalized separation of CO_2_ generation via decarboxylation of C4 compounds and subsequent CO_2_ fixation by Rubisco [25], [26]. In these plant cells, PPDK is located in chloroplasts where it converts pyruvate to PEP. The PEP is then transported to the cytosol where it is carboxylated (using HCO_3_^−^) via PEPC. The C4 acids produced diffuse to the proximal part of the cell where they are decarboxylated in the mitochondria by NAD-malic enzyme. The resulting CO_2_ may enter the chloroplasts where it is captured by Rubisco.

Both sequenced diatoms possess two malic enzymes that decarboxylate malate to pyruvate. An NADP-malic enzyme has been proposed for diatoms by Granum et al. [3]. A potential NAD-dependent malic enzyme (56501) was also identified that is predicted to be localized to mitochondria and displays sequence similarity to malic enzymes from the C4 plants *Amaranthus hypochondriacus* [25], *B. cycloptera* and *B. aralocaspica* [25]. The dinucleotide binding site of the malic enzyme from *A*. *hypochondriacus*, *P. tricornutum* and *T. pseudonana* possesses a similar amino acid composition suggesting that NAD is the preferred co-factor. These data suggest that in diatoms, decarboxylation of malate to generate CO_2_ may occur within mitochondria, which are often closely associated with plastids. It is important to note however, that any CO_2_ molecules released from the mitochondria must cross six membranes to enter the plastid stroma. Moreover, it is likely that CO_2_ would be converted to HCO_3_^−^ by CA activity during movement between the mitochondria and plastids, thereby reducing at least part of the elevated CO_2_ concentration. In this case, the C4 pathway would become a futile cycle whereby HCO_3_^−^ is first fixed and then formed again, thereby dissipating ATP for PEP formation.

Co-occurrence of PEPCK- and malic enzyme-based decarboxylation pathways in the same organism was also observed in the C4-plant *Urochloa panicoides* [27], [28]. Apparently, in diatoms both enzymes may contribute to the decarboxylation of the C4-acid. In some of the higher plants which perform C4 metabolism, the pyruvate formed by decarboxylation of malate, using the NAD-malic enzyme, can be used for amino acid synthesis (Fig. 1) [28] or oxaloacetate formation thereby replenishing mitochondrial pools of C4 acids. Oxaloacetate can also be oxidized in the TCA cycle. Decarboxylation of OAA by PEPCK generates PEP, which can be used for gluconeogenesis or be transformed into pyruvate by a pyruvate kinase (Fig. 1). The presence of a plastid-targeted putative PEP-transporter TPT1 (24610) and the plastid targeted PPDK (21988) suggests that pyruvate is phosphorylated inside the plastid to produce PEP. PEP can be used to produce aromatic amino acids (Shikimate pathway) and lipids or can be exported to provide the acceptor molecule for HCO_3_^−^ fixation by PEPC (Fig. 1). This pathway is also known from C4 plants that decarboxylate PEP inside the mitochondria (see [25], [28]–[29]). However, most C4-plants carry out the first carboxylation step in the cytosol [29]. In *P. tricornutum* and *T. pseudonana* PEP might be exported from the plastid and carboxylated at the ER/CER/PPC by PEPC1 leading to a micro-compartmentalization of the enzyme to the outer chloroplast membranes. Lee and Kugrens [30] have speculated that the evolutionary success of heterokonts might be partly attributed to the use of the periplastidic space as an acidic generator of CO_2_ from bicarbonate.

In summary, the accumulated evidence indicates that a functioning C4 pathway in diatoms (Fig. 1) requires a spatial separation between CO_2_ production via decarboxylation of OAA and malate in mitochondria, and CO_2_ utilization by Rubisco in the plastid. Furthermore, localization of the first carboxylation step by PEPC is still unclear. The energetic cost of a futile cycle raises the possibility that the C4 metabolism may help cells dissipate excess light energy, a pathway that would presumably require down-regulation under energy-limiting conditions. This suggests that the flow of metabolites in this pathway would be affected by light intensity. Localization of key enzymes and determination of expression of C4 related genes in cells exposed to low levels of CO_2_ could shed light on this issue.

Two lines of evidence support the hypothesis that a biophysical CCM operates in diatoms, as in many other aquatic photosynthetic organisms [7], [31]–[33]. First, we identified in the genome of *P. tricornutum* three genes that encode different systems for bicarbonate uptake. One protein (45656) shows homology to sodium/bicarbonate transporters in various organisms and appears to be localized to the plastid. A second protein (32359) is homologous to sodium-dependent anion exchangers and also possesses the bipartite presequence for plastid targeting. The third protein (54405) shows similarity to Cl^−^/HCO_3_^−^ exchangers abundant in red blood cells. *T. pseudonana* appears to possess a single sodium/bicarbonate transporter (24021) that is targeted to the plastid. The hypothesis resulting from these observations is that inhibitors shown to prevent HCO_3_^−^ uptake as shown in *Ulva* sp. [34], should inhibit uptake of HCO_3_^−^ and thereby the rate of photosynthesis in both diatoms.

The second form of support for a biophysical CCM is identification of numerous genes encoding carbonic anhydrases (CA) in the diatom genomes. The overall sequence similarities among CAs are rather low and they are commonly identified by the presence of conserved domains and by their biochemical properties [35]. Seven CAs are predicted for *P. tricornutum*. Two CAs (51305, 45443) are related to the beta type and show similarity to CAs found in both plants and prokaryotes. Based on the presence of a plastid targeting presequence and physiological experiments [36], one of these proteins (51305) is located in the plastid as has been demonstrated by GFP fusion proteins [37]. The other (45443) has a signal peptide. The five other identified CAs (35370, 44526, 55029, 54251, 42574) apparently belong to the alpha family and all possess signal peptides. A recent study by Szabo and Colman [38] provided experimental evidence for the presence of CA in the periplasmic space of *P. tricornutum* suggesting that at least a subset of the signal peptide-possessing CAs are likely targeted to the periplasmic space. Surprisingly, similarity of the CAs between the two diatoms *T. pseudonana* and *P. tricornutum* is rather low. *T. pseudonana* appears to possess more intracellular CAs without signal and transit peptides than *P. tricornutum.* One exception is the carbonic anhydrase 22391 from *T. pseudonana* which also possesses a signal peptide. Whether this CA is secreted or targeted to ER or periplastidic space remains to be investigated, while it seems clear that it is not plastid localized. The difference between the two diatoms may indicate specialization of the enzyme depending on different ecological niches. This is supported by recent findings that expression of beta carbonic anhydrases may be regulated by several factors including CO_2_ and light [39].

Despite the extensive *in silico* analyses described here, the potential mechanism by which CO_2_ is delivered to Rubisco remains elusive. In the well-studied green alga, *Chlamydomonas*, a thylakoid-located alpha CA facilitates conversion of HCO_3_^−^ to CO_2_, thereby raising its concentration in close proximity to Rubisco [40]. Mutants impaired in this CA demand high CO_2_ concentrations for growth (see [7], [31]). In *P. tricornutum*, the plastid-localized CA (51305) is a beta type CA rather than an alpha type. However, it too localizes to the thylakoids where it forms particles, most probably close to the girdle lamellae [37], and its expression is strongly enhanced under low CO_2_ conditions by a mechanism involving cAMP [41]. The other beta type CA (45443) is probably located in the ER or the periplasmic space and is constitutively expressed even under high CO_2_ concentrations [42]. The presence of bicarbonate transporters in the chloroplast envelope (Fig. 1) is consistent with the operation of a biophysical CCM but is not essential for a C4-like CCM, where the initial HCO_3_^−^ fixation occurs outside the plastid. Finally, enhanced uptake of inorganic carbon, both as CO_2_ or HCO_3_^−^, would be consistent with both types of CCM, whereas raising the concentration of C_i_ within the cells [43] is more consistent with the biophysical CCM. The high affinity of PEPC for HCO_3_^−^ would be expected to alleviate the need to accumulate high C_i_ concentrations internally. Induction of an extracellular CA at low CO_2_ concentrations was also observed in *P. tricornutum*. Such a CA would be expected to facilitate the rate of CO_2_ formation in the unstirred layer surrounding the cells and thereby to supply CO_2_ for photosynthesis by either CCM type. In summary, clear evidence that supports either CCM mode as the *only* way to raise the CO_2_ concentration in close proximity of Rubisco is presently missing due to lack of sufficient biochemical evidence.

### Photorespiration and glyoxylate metabolism

Photorespiration is the inevitable consequence of the ability of either CO_2_ or O_2_ to cleave the double bond obtained in RuBP after enolization by Rubisco. In higher plants, photorespiration is thought to provide the photosynthetic machinery with some protection against photoinhibition [44]–[47]. Mutants of tobacco (*Nicotiana tabacum* L.) defective in enzymes of the photorespiratory pathway demonstrated enhanced photoinhibition under high light conditions [48], [49]. The specificity factor (τ) of Rubisco, a measure of its ability to discriminate CO_2_ from O_2_, is considerably higher in diatoms than in cyanobacteria and green algae (reviewed in [50]) suggesting a lower rate of O_2_ fixation in diatoms than observed in members of the green lineage. This is supported by studies showing photorespiratory activity in diatoms at a reduced rate than expected from studies with higher plants [51]–[54].

**Figure 2:**
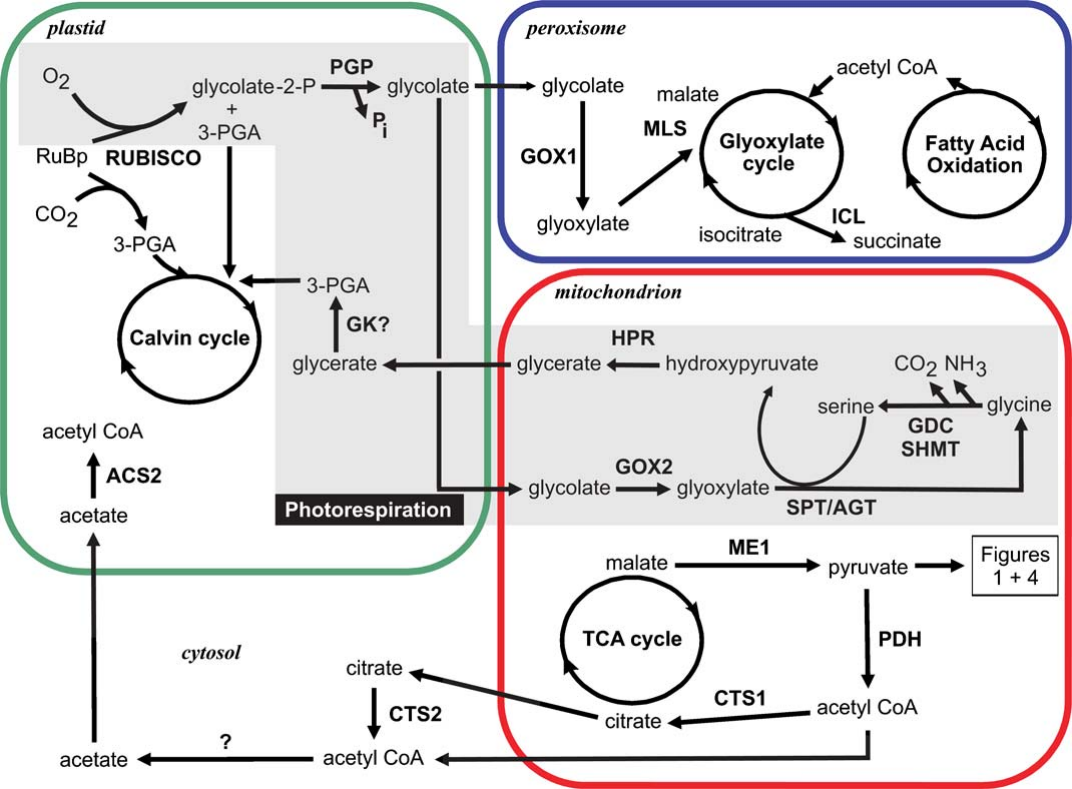
Model for photorespiration and associated pathways in diatoms based on the annotations of the *Phaeodactylum tricornutum* and *Thalassiosira pseudonana* genomes. For simplicity, the number of oragenelle membranes has been reduced in this figure. A gene model for glycerate kinase (GK) could not be found in either genome. The bacterial-type glyoxylate to glycerate metabolism is not shown due to uncertainty in the localization of the enzymes. Enzyme Abbreviations: ACS: acetyl CoA synthetase; CTS: citrate synthase; GDC: glycine decarboxylase; GOX: glycolate oxidase; GK: glycerate kinase; HPR: hydroxypyruvate reductase /glycerate dehydrogenase; ICL: isocitrate lyase; ME1: NAD malic enzyme; MLS: malate synthase; PDH: pyruvate dehydrogenase; PGP: 2-phosphoglycolate phosphatase; RUBISCO: ribulose-1,5-bisphosphate carboxylase; SHMT: serine hydroxymethyltransferase; SPT/AGT: serine-pyruvate/alanine-glyoxylate aminotransferase.

When O_2_ out-competes CO_2_ for RuBP, one molecule of 2-P-glycolate and one molecule of 3-P-glycerate are produced. The latter may enter the Calvin cycle, whereas 2-P-glycolate, a metabolite known to inhibit the Calvin cycle enzyme triosephosphate isomerase [55] must be degraded via the photorespiratory pathway (see Fig. 2). In higher plants, metabolism of 2-P-glycolate takes place via the C2 cycle [56]. Following cleavage of the phosphate group, glycolate is exported out of the chloroplast and enters the peroxisome where it is oxidized to glyoxylate, followed by transamination to form glycine which enters the mitochondrion. The glycine decarboxylase complex together with serine hydroxymethyltransferase, catalyzes the condensation of two glycines to one serine with the consequent release of ammonium ion and CO_2_. The serine is further metabolized back to P-glycerate which may then enter the Calvin cycle in the chloroplast. Thus, out of four carbons entering the C2 cycle, three are converted back to PGA and one is released in the form of CO_2_.

In microalgae, the cyclic process of PGA recovery is not well-studied. More often, glycolate metabolism is studied in the context of its excretion as a waste-product to circumvent unfavourable growth conditions [57]–[60]. A large fraction of glycolate produced by fixation of O_2_ is released from the cell [61]–[63] and may serve as an important source of organic carbon in the water body. Leboulanger et al. [64] found high concentrations of glycolate in seawater at both oligotrophic and eutrophic sites, suggesting that photorespiration may be ubiquitous in the marine environment. Photorespiration may therefore represent an important loss of fixed carbon, either via released glycolate or CO_2_. Recent studies on photorespiration in the cyanobacterium *Synechocystis* sp. PCC 6803 showed considerable rates of glycolate formation even when the cells were exposed to high levels of CO_2_ such as 5% CO_2_ in air [65]. Interestingly in diatoms, when *T. weissflogii* and *T. pseudonana* were exposed to ^14^CO_2_ for 5 sec, a considerable label (15%) was detected in glycolate in *T. pseudonana* but only 5% in *T. weissflogii* [5]. This may indicate a higher level of CO_2_ in the vicinity of Rubisco in the case of *T. weissflogii* and consequently a reduced oxygenase activity.

Recent studies on the expression of key genes in the C2 cycle in *Thalassiosira* sp. suggest the photorespiratory pathway is active in diatoms and plays a critical role in carbon and nitrogen metabolism in the cell [63], [66]. We have identified most of the enzymes likely involved in a C2-type glycolate pathway in the genomes of *P. tricornutum* and *T. pseudonana* (Fig. 2). The annotation of enzymes in the C2 pathway confirms several differences between the photorespiratory cycle in diatoms and in higher plants, and corroborates the scheme proposed by [67]. In algae, it has been suggested that two types of glycolate-oxidizing enzymes exist: a glycolate oxidase in Chrysophyceae, Eustigmophyceae, Raphidophyceae, Xanthophyceae and Rhodophyceae, and a glycolate dehydrogenase in Chlorophyceae, Prasinophyceae, Cryptophyceae and Bacillariophyceae [68]. Winkler and Stabenau [67] further suggest that in diatoms glyoxylate is synthesized via a glycolate dehydrogenase in both peroxisomes and mitochondria. In both *P. tricornutum* and *T. pseudonana*, two proteins similar to glycolate oxidases (GOX) or possibly glycolate dehydrogenases (GDH) were identified: one of the two proteins (22568) contains the peroxisomal targeting motif PTS1, which is common in many eukaryotes and characterized by the consensus sequence (S/C/A)(K/R/H)(L/M) located at the extreme carboxy-terminus [69]. The other protein (50804) appears to be targeted to the mitochondria. This suggests that at least one glycolate oxidizing enzyme in each diatom is localized in the peroxisome, although based on the previous biochemical studies of Suzuki et al. [68] and Winkler and Stabenau [67], it is unclear whether it catalyzes production of hydrogen peroxide. Moreover, the potential for peroxisomal activity is corroborated by identification of a malate synthase (54478). In both diatoms, the enzymes for serine synthesis and metabolism have been found targeted to the mitochondria: serine-pyruvate/alanine-glyoxylate aminotransferase (SPT/AGT, 49601), glycine decarboxylase and serine hydroxymethyltransferase GDC/SHMT (56477, 22187, 32847, 18665, 17456), hydroxypyruvate reductase (56499) (Fig. 2). Interestingly, we were not able to identify a gene for glycerate kinase in *P. tricornutum* or in *T. pseudonana*. This enzyme catalyzes the last reaction of the C2 cycle and appears to be present in cyanobacteria, the green algal lineage, the red algal lineage, but only sporadically in alveolates and heterokonts. The absence of this enzyme poses the question of how, or whether, glycerate can be transformed into 3-P-glycerate to be reintegrated into the Calvin cycle. An alternative to glycerate, and thereby 3-P-glycerate, as the endpoint of photorespiration is the possibility that all the glycine and serine produced from the fixation of oxygen are instead shunted to other pathways. For example, the formation of the antioxidant glutathione from photorespiratory glycine has been previously demonstrated (reviewed in [70]).

Another pathway for glyoxylate metabolism, the tartronate semialdehyde pathway, is known in cyanobacteria [70]. *Synechocystis* mutants were used to illustrate that a C2 pathway and glyoxylate/glycerate pathway (via glyoxylate carboligase and tartronic semialdehyde reductase) cooperate in the metabolism of 2-phosphoglycolate [65]. Genes encoding a putative tartronate semialdehyde reductase (45141) and a putative glyoxylate carboligase, also called tartronate semialdehyde synthase (56476), have been found in both diatoms. The closest BLAST matches to the models for tartronate semialdehyde reductase are genes encoding 3-hydroxyisobutyrate dehydrogenases. The two enzymes are part of the same enzyme family, making a definitive assignment difficult. The putative *P. tricornumtum* tartronate semialdehyde reductase (45141) has a mitochondrial targeting peptide while the targeting for the *T. pseudonana* model (2669) is unclear. The closest BLAST matches to the predicted glyoxylate carboligase were acetolactate synthase, however, these two enzymes are also closely related and difficult to distinguish. Both predicted carboligases appear to have chloroplast transit peptides, but the evidence is weak and therefore targeting of glyoxylate carboligase remains uncertain in both diatoms. The presence of glyoxylate metabolism is supported by an early study of Paul and Volcani [51] showing that the activity of glyoxylate carboligase in the diatom *Cylindrotheca fusiformis* is affected by light intensity. These data suggest that similar to cyanobacteria, diatoms combine C2 and glyoxylate/glycerate pathways to metabolize 2-phosphoglycolate back to the Calvin cycle.

### Reductive/oxidative pentose phosphate pathway

Photosynthetic carbon fixation in plants and algae is performed by the Calvin cycle. Some Calvin cycle enzymes in land plants are of cyanobacterial origin, while others have been replaced by protobacterial or eubacterial enzymes [71]. Carbon fixation in land plant plastids is highly regulated, either by substrates and ions like Mg^2+^ or by light-dependent redox regulation either at the transcriptional [72], [73] or the enzymatic level via the ferredoxin/thioredoxin-system [74], [75]. In addition to the Calvin cycle (reductive pentose phosphate pathway), plastids from land plants and green algae possess an oxidative pentose phosphate pathway (OPP). This ubiquitous process produces NADPH and pentose-phosphates for biosynthesis of nucleotides, amino acids and fatty acids in the dark by decarboxylation of glucose-6-phosphate.

**Figure 3:**
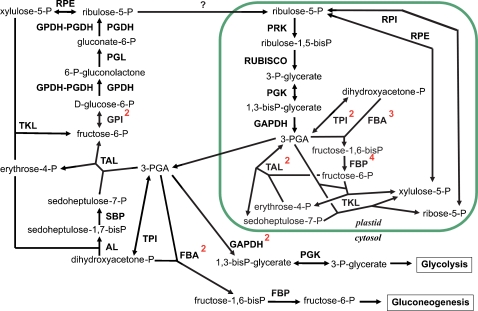
Model of the oxidative and reductive pentose phosphate pathways and related reactions in *P. tricornutum*. For simplicity, the number of organelle membranes has been reduced in this figure. The superscript numbers attached to the enzyme names indicate the number of isoenzymes within the respective compartment. Enzyme abbreviations: AL: aldolase; FBA: fructose-1,6-bisphosphate aldolase; FBP: fructose-1,6-bisphosphatase; GAPDH: glyceraldehyde-3-phosphate dehydrogenase; GPI: Glucose-6-phosphate isomerase; GPDH: glucose-6-phosphate dehydrogenase; PGL: phosphor-gluconate lactonase; PRK: Phosphoribulokinase; RUBISCO: ribulose-1,5-bisphosphate carboxylase; PGDH: 6-phospho-gluconolactone dehydrogenase, PGK: phospho-glycerate kinase; RPI: ribose-5-phosphate isomerase; RPE: ribulose-phosphate epimerase; RPI: ribose-5-phosphate isomerase; TKL: transketolase; TAL: transaldolase; TPI: triose-phosphate isomerase; SBP: seduheptulose-1,7-bisphosphatase.

As both pathways in plastids are interconnected, operating them simultaneously would result in a futile cycle, using up energy in the form of ATP without net CO_2_ fixation. Thus in plastids of land plants and green algae some of the enzymes of the Calvin cycle like the phosphoribulokinase (PRK), glyceraldehyde-3-phosphate dehydrogenase (GAP-DH), fructose-1,6-bisphosphatase (FBP), and seduheptulose-1,7-bisphosphatase (SBP) are activated in the light via reduction by thioredoxin (and become inactive in the dark), while the key enzyme of the OPP, the glucose-6-phosphate dehydrogenase (G6PDH) is active in the dark, but inhibited after reduction in the light. In contrast to higher plants, there is apparently no complete oxidative pentose phosphate pathway (OPP) in the plastids of several diatoms [9], [10] as well as in *P. tricornutum*, suggesting diatom plastids in general lack this pathway. Two putative 6-phosphoglucono-lactonases might be targeted to the cytosol (31882) and to the plastid (38631), however, both genes are not yet supported by ESTs. The other two required enzymes glucose-6-phosphate dehydrogenase (G6PDH, 30040, and the G6PDH component of a G6PDH/6PGDH fusion protein, 54663) and 6-phosphogluconate dehydrogenase (6PGDH, 26934, and the 6PGDH component of the G6PDH/6PGDH fusion protein, 54663) were found to be cytosolic enzymes, indicating that the complete OPP is only functional in the cytosol (Fig. 3).

The only Calvin cycle enzymes encoded on the plastid genome are the small and the large subunit of the Rubisco (GenBank AY819643) [76]. All other genes encoding primary enzymes of the Calvin cycle have been identified in the nuclear genomes of *P. tricornutum* and *T. pseudonana* (see Fig. 3). The only exception is the gene for the sedoheptulose bisphosphatase (SBP). The only SBP gene we identified (56467) encodes a protein that lacks a plastid targeting sequence and thus appears to be localized within the cytosol. This SBP, however, is not contained in the large set of ESTs from *P. tricornutum*, indicating that it is not actively transcribed under the applied conditions. SBP catalyses the reaction from sedoheptulose-1,7-bisphosphate to sedoheptulose-7-phosphate in the Calvin cycle; it is unclear yet whether the SBP reaction does not occur in diatom plastids or - more likely - whether this reaction is performed by one of the plastidic FBPs as shown for FBP I in cyanobacteria [77]. Interestingly there is a gene encoding a plastidic FBP (FBPC2, 42456) with a bipartite plastid targeting presequence that is located about 500 bases upstream of the SBP gene in the same orientation (similar as in *T. pseudonana*). There is a theoretical possibility that both genes might be transcribed together and - after excision of a putative intron - might be translated as a fusion protein, thus the SBPase could be imported in a piggy-back manner, although this hypothesis has not yet been supported by transcript analyses (Weber and Kroth, unpublished).

Genes encoding the Calvin cycle enzymes fructose-1,6-bisphosphate aldolase (FBA) and FBP are present in several copies. There are two class II aldolases (22993, bd825) and one class I (24113) aldolase in the plastids, while a class I (42447) and a class II (29014) aldolase are found in the cytosol. Four plastidic FBPases have been identified (FBPC1: 42886; FBPC2: 42456; FBPC3: 31451; FBPC4: 54279) and one cytosolic enzyme (23247). The redundancy of isoenzymes may partially reflect the evolution of diatoms by secondary endocytobiosis [15], [75]. Some of the isogenes may have either a cyanobacterial or a rhodophytic origin or are related to respective enzymes from oomycetes. Other genes may also have been transferred by lateral gene transfer from bacteria or have been duplicated within the heterokonts [78].

Redox-regulation of enzymatic activity is critical for plastid functions. Thioredoxin is a small protein that is reversibly reduced in the light by ferredoxin/thioredoxin reductase (FTR) and is able to reduce target enzymes resulting in altered enzymatic activities [75]. Several genes encoding thioredoxins (Trx) were identified in *P. tricornutum*, including the genes for Trxs f (46280) and m (51357) both possessing typical plastid targeting signals. Three genes encoding Trx h proteins (48539, 56471, 48141/56521) were identified, one of which (48539 plus possibly 48141/56521) contains a presequence for targeting into ER/periplastidic space (respective homologues are also found in *T. pseudonana*). This is surprising because Trx h is located in the cytosol in all other organisms examined so far. Genes encoding two plastidic Trxs y (33356, 43384), a mitochondrial Trx o (31720) and a ferredoxin-thioredoxin oxidoreductase (50907, needed for Trx reduction) were also identified. These results imply that thioredoxin based light-regulation is functional in diatom plastids, although far fewer plastid enzymes in diatoms than in plants may be actual Trx targets (see [8]).

Another group of proteins involved in redox-regulation in land plant plastids are glutaredoxins (Glrx), which are involved in fine-tuning of the thioredoxin system [79]. In *P. tricornutum* we predict two glutaredoxins to be targeted into the plastids (43497, 39133), one to the cytosol (16854), and one to the mitochondria (37615). Similar to the unusual periplastidal/ER associated Trxs h (48539/48141), one glutaredoxin (56497) also contains a presequence for targeting into ER/periplastidic space. Taken together, thioredoxins and glutaredoxins are present in the mitochondria, plastids and cytosol of *P. tricornutum* and *T. pseudonana*, although their functionality and specificity is unclear.

The plastidic fructose-bisphosphatase (FBP) is the only enzyme in diatoms for which there is direct evidence of redox-regulation by thioredoxin [9]. The PRK also possesses the conserved cysteines for redox regulation, although due to a shift of the redox midpoint potential of this enzyme, it does not get oxidized *in vivo* and thus is permanently active [9]. Diatom plastids also possess a different GADPH enzyme compared to green algae and land plants, termed GapC1 (25308), which does not contain the respective cysteines [80] and which is not affected by oxidation or reduction (Michels, A. Wedel, N., and Kroth, P.G., unpublished). The chloroplast ATPase in land plants is modulated by thioredoxin by lowering the energy threshold of the membrane potential necessary to activate the enzyme. The sequence cassette on the γ subunit containing the necessary cysteines (AtpC, 20657) in land plants is missing in diatoms as well as in red algae.

Other plastidic enzymes which are affected by thioredoxin in land plants are not found in *P. tricornutum* or *T. pseudonana*. (i) In land plants and in green algae there are two malate dehydrogenases, one of which is NAD-dependent and one of which is NADP-dependent. The NADP-dependent enzyme is redox-regulated via thioredoxin and serves as a valve for excess NADPH [81]. Based on enzymatic and *in silico* analyses, the redox-regulated isoenzyme appears to be missing from diatom plastids (Mertens and Kroth, unpublished). (ii) ADP-glucose pyrophosphorylase (AGPase) in land plant plastids produces ADP-glucose, the substrate for starch synthesis [82]. Diatoms do not possess a plastidic AGPase, which is consistent with the fact that they export all carbohydrates immediately from the plastids and store them as chrysolaminaran in cytosolic vacuoles. (iii) The Rubisco activase responsible for activation of Rubisco [83], is apparently also missing from diatom plastids as no gene for this protein has been found in the genomes of *P. tricornutum* or *T. pseudonana*. (iv) Another system regulating the Calvin cycle in land plants is the formation of enzyme complexes of GAPDH and PRK by the small protein CP12 via disulfide bridges [84]. In land plants and in green algae these complexes form in the dark, and in the light they are reduced by thioredoxin in the presence of NADPH, dissociate and release GAPDH and PRK activity [85]. A comparison of native GAPDH and PRK enzymes from stromal extracts of diatoms and land plants by gel filtration revealed that diatoms do not form GAPDH/PRK/CP12 complexes (Michels, Wedel and Kroth, in preparation), accordingly we were not able to identify genes for putative CP12 proteins in diatom genomes.

Interestingly, during our genome analysis we identified a few cases of unusual gene fusions. When transcribed as a single mRNA they may form fusion proteins consisting of two metabolic enzymes that are connected by spacers of 8 to 25 amino acids. We found three metabolic enzyme pairs that apparently are fused to each other because they are transcribed by a single mRNA: the mitochondrial triosephosphate-isomerase/glyceraldehyde-3-phosphate dehydrogenase (TIM-GAPC3, 25308) [80], a cytosolic UDP-glucose-pyrophosporylase/phosphoglucomutase (UGP/PGM, 50444), and a cytosolic glucose-6-phosphate-dehydrogenase/6-phosphogluconate-dehydrogenase (G6PDH/6PGDH, 54663) fusion protein. The fact that each pair of enzymes catalyzes two subsequent metabolic reactions indicates that fusing these genes may result either in a better regulation or a faster conversion of substrates. However, there is evidence that at least some of these fusion proteins may be cleaved post-translationally [80] (Majeed and Kroth, unpublished). Interestingly, the TIM-GAPDH (present in *T.pseudonana* and *P. tricornutum*) and the UGP/PGM are found in the genomes of the stramenopiles *Phytophthora ramorum* and *Phytophthora sojae*, while the G6PDH/6PGDH is not.

### Glycolysis

**Figure 4:**
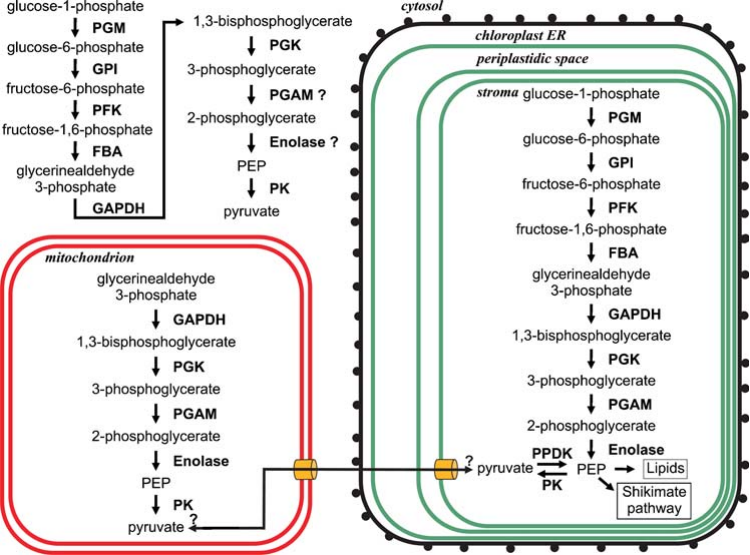
Model of the glycolytic reactions in the cytosol and related pathways within mitochondria and plastids of *P. tricornutum*. Enzyme abbreviations: PGM: phosphoglucomutase; GPI: Glucose-6-phosphate isomerase; PFK: Phosphofructokinase; FBA: fructose-1,6-bisphosphate aldolase; GAPDH: glyceraldehyde-phosphate dehydrogenase; PGK phospho-glycerate kinase; PGAM: phosphor-glycerate mutase; PK: pyruvate kinase; PPDK: pyruvate-phosphate dikinase.

Glycolysis is a universal cytosolic pathway for degradation of hexoses and results in pyruvate, which may be targeted to the mitochondria in eukaryotic organisms performing aerobic degradation or may be utilized in various other ways in organisms capable of living in anaerobic conditions. Several enzymes involved in glycolysis occur as a number of isoenzymes in *P. tricornutum* and *T. pseudonana.* For instance there are five genes for phosphoglucomutases (PGM) present in the *P. tricornutum* genome: two of the gene products (48819, 50718) are likely to be targeted to the plastid while the other isoenzymes apparently are located in the cytosol (51225, 50118 and the PGM component of a UDP-Glucose-Pyrophosphorylase/Phosphoglucomutase fusion protein 50444). Similarly there are three phosphoglycerate kinases predicted to be targeted either to the cytosol (51125), the mitochondria (48983) or the plastid (29157). Recent analyses using GAPDH genes from diatoms and other organisms indicate a common origin of all chromalveolates (86). Of the six identified GAPDH enzymes in *P. tricornutum*, two are targeted to the mitochondria (32747 and the GapC3 component of a TPI/GapC3 fusion protein 25308) and one is targeted to the plastids (22122) [80]. GapC2, assigned to be cytosolic [80] is present in two copies encoded in the same orientation on chromosome 16, with a distance of approx. 24 kilo base pairs (51128, 51129). A third cytosolic GAPDH enzyme was additionally identified (23598). Three genes for glucose-6-phosphate isomerases (GPI) were found, encoding a plastidic GPI (56512) and two cytosolic enzymes [87] with genes located next to each other in opposite direction (23924, 53878). We found only genes encoding a plastidic (56468) and two mitochondrial enolases (bd1572, and the apparently unfunctional bd1874) in *P. tricornutum.* However, in *T. pseudonana* a cytosolic and a mitochondrial enolase have been found (40771, 40391). This indicates that all reactions of the glycolysis may potentially occur within the plastid (Fig. 4), where some of them simply represent essential enzymes of the Calvin cycle. Also surprising is the fact that there are isoenzymes of the complete second half of the glycolysis possessing mitochondrial presequences (see Fig. 4). In some cases the respective enzymes have been shown to be targeted into the mitochondria by fusing the presequences to GFP (C. Rio Bartulos, personal communication). Similarly the translocation of glycolytic reactions to other organelles has been described in unicellular green algae [88].

No gene encoding a hexokinase for phosphorylation of glucose was detectable in either *P. tricornutum* or *T. pseudonana*. Instead, genes for glucokinases were detected in both species. This observation conforms to the trend that sugar-specific kinases are typical in prokaryotes and unicellular eukaryotes, whereas hexokinases with broader substrate specificities are typical in multicellular eukaryotes [89]. The *P. tricornutum* cytosolic glucokinase (48774) might additionally be involved in the chrysolaminaran pathways (see below).

### Storage products–synthesis and degradation

Chrysolaminaran is the principal energy storage polysaccharide of diatoms. The relatively high contribution of chrysolaminaran to marine particulate matter underscores this molecule's significant role in the oceanic cycling of carbon [90]–[92]. It generally comprises between 10 and 20% of the total cellular carbon in exponentially growing diatoms but can accumulate to up to 80% of the total cellular carbon in cells whose growth is limited by nitrogen [93]. Chrysolaminaran concentrations undergo a diel rhythm characteristic of an assimilatory and respiratory product, accumulating during the daylight and becoming depleted in the dark [90], [94], [95]. The structure of chrysolaminaran is fundamentally based on a β-1,3-linked glucan backbone, which is infrequently branched with mainly β-1,6-linkages [96]–[102]. Vacuolar localization of chrysolaminaran in several diatom species, including *P. tricornutum* and *T. pseudonana*, was demonstrated by staining with aniline blue [103] and by immunolabeling with a monoclonal anti-1,3-β-D-glucan antibody [99].

The biochemical pathways leading to chrysolaminaran synthesis and degradation have not been elucidated. However, enzyme assays of cell-free extracts from the diatom *Cyclotella cryptica* demonstrated that the formation rate of UDP-glucose was ≥20-fold greater than for any other nucleoside-diphosphate-glucose and that UDP-glucose served as a substrate for chrysolaminaran synthesis [104]. Furthermore, exo-1,3-β-glucanase activity was detected in several planktonic diatoms and upregulation of this activity coincided with chrysolaminaran degradation in the diatom *Skeletonema costatum* [94].

We focused on exo- and endo-1,3-β-glucanases and β-glucosidases as the primary enzymes involved in digesting chrysolaminaran. We found four putative exo-1,3-β-glucanases in *P. tricornutum*, all belonging to the glycosyl hydrolase family 16 (49294, 56510, 56506, 49610). All orthologues possess an N-terminal signal peptide, except (49610), and all contain a C-terminal transmembrane helix. In addition, one (56510) possesses a putative C-terminal ER-retention signal (REEL). Of the four exo-1,3-β-glucanases, only one (49294) was represented in *T. pseudonana* (13556). Three putative endo-1,3-β-glucanases were identified in *P. tricornutum*, two belonging to glycosyl hydrolase family 16 (54681, 54973) and one to family 81 (46976). One of these (54681) consists of 1028 amino acid residues and has both an N-terminal signal peptide and a C-terminal transmembrane domain. The second (54973) has a signal peptide but no transmembrane helix and it is only about half the length. Curiously, the third enzyme (46976) apparently lacks a signal peptide but has an N-terminal transmembrane helix, making it a candidate for a type II transmembrane protein. Among the sequences, 54681 from *P. tricornutum* and 35711 from *T. pseudonana* are most similar to each other. In a ClustalW tree of endo-β-glucanases, these sequences clustered with bacterial endoglucanases (*Rhodothermos marinus*, *Bacillus circulans*, and *Sinorhizobium meliloti*), and (54973) only very weakly associated with these. The family-81 endoglucanases from the two diatom species grouped together but were apparently still relatively divergent, the next closest sequences being a pair of family-81 endoglucanases from *Arabidopsis thaliana*.

Three putative β-glucosidases were identified in *P. tricornutum*, one belonging to glycosyl hydrolase family 1 (50351) and the other two (45128, 49793) to family 3. In *T. pseudonana*, only a single β-glucosidase was identified (28413), and this belonged to glycosyl hydrolase family 1. In addition to a signal anchor (50351), only one of the *P. tricornutum* family-3 β-glucosidases (45128) appears to have a C-terminal transmembrane helix. All three *P. tricornutum* orthologues were represented by ESTs, but to varying degrees.

Overall, at least 10 enzymes predicted to digest 1,3-β-glucans were identified in *P. tricornutum*. Presumably, at least one of the exo-1,3-β-glucanases and one of the endo-1,3-β-glucanases act complementarily to digest the principle β-1,3-linkages of chrysolaminaran. The products of efficient digestion by this suite of enzymes would be primarily free glucose, with relatively small amounts of glucosyl oligosaccharides dominated by β-1,6-linkages (e.g. gentiobiose) derived from surviving chrysolaminaran branch points. A β-glucosidase could hydrolyze such oligosaccharides to free glucose. The free glucose generated from complete chrysolaminaran degradation would subsequently be phosphorylated by glucokinase.

The vacuolar localization of chrysolaminaran implies that the degradative enzymes are also localized there. However, the exo-1,3-β-glucanase (56510) possesses a C-terminal ER-retention signal in addition to the signal peptide and C-terminal transmembrane helix. In yeast, transmembrane domains can serve as localization signals for sorting proteins from the ER, with the destination (plasma membrane or vacuole) dependent upon transmembrane helix length and composition rather than on a specified sequence [105]. We identified one gene for a glucokinase in *P. tricornutum*. As described above we conclude the enzyme to be involved in the cytosolic glycolysis (48774). Although there is no EST support, it is possible that by intron splicing the glucokinase may possess a signal peptide, which might allow targeting to the vacuole (compare to 56514). Interestingly, similar to the glucanases the enzyme possesses a C-terminal transmembrane helix, indicating that it might be integrated into membranes as shown for various hexokinases from plants [106]. In addition to a number of bacterial sequences, the most similar sequence to the diatom glucokinases is the glucokinase of *Cyanidioschyzon merolae*. This enzyme apparently also lacks a hexokinase and its glucokinase also contains a C-terminal transmembrane helix [107]. The simplest model is that the diatom glucan-digesting enzymes and the glucokinase are anchored at their C-termini to cytosolic membranes like the vacuolar membrane either being oriented towards the cytosol or to the vacuole. The localizations of the β-glucosidases are heterogeneous, however, and for any one of them to serve as a vacuolar gentiobiase would require localization by mechanisms other than those that localize the β-glucanases or the glucokinase.

The hypothesis proposed here for degradation of chrysolaminaran has implications for the generation of glucosyl phosphate intermediates from an energy storage glucan. First, enzymes responsible for chrysolaminaran degradation apparently were recruited during evolution from enzymes normally associated with extracellular polysaccharides. Second, and as a consequence, degradative and phosphorylating steps are decoupled in diatoms. In organisms that metabolize starch or glycogen, the degradative and phosphorylating steps are achieved either concomitantly by an ATP-independent pathway or separately by an ATP-dependent pathway in which phosphorylation is catalyzed by hexokinase (for reviews, see [108], [109]. The apparent occurrence of only glucokinase in both *P. tricornutum* and *T. pseudonana* may, apart from reflecting their evolutionary heritage, be an adaptation to a dedicated ATP-dependent pathway for chrysolaminaran digestion. Bacterial glucokinases, such as those of *Escherichia coli*, *Zymomonas mobilis*, *Bacillus stearothermophilus*, and *Streptococcus mutans*, have a high specificity and moderately high but relatively narrow K_M_ range for glucose (K_M_ = 0.22–0.61 mM; [99]–[102]) compared with broad-specificity eukaryotic hexokinases (K_M_ = 0.007–2.5 mM; [89], [110]. In diatoms, such a glucokinase could cope with substantial fluxes in glucose concentrations and ensure that the phosphorylation is efficient as high concentrations of free glucose are liberated during chrysolaminaran degradation. The affinities and kinetics of the diatom glucokinases will need to be characterized, to assess the validity of this hypothesis.

The synthetic pathway of chrysolaminaran is essentially unknown. Based on enzyme activity assays of *C. cryptica*, UDP-glucose likely serves as the substrate for chrysolaminaran synthesis [104]. The apparent absence of genes encoding ADP-glucose pyrophosphorylase in either diatom species provides further support that UDP-glucose serves as the substrate for chrysolaminaran synthesis. Interestingly, the UDP-glucosyl pyrophosphorylase (UGP) from *C. cryptica* was not inhibited by 3-P-glycerate or inorganic phosphate, suggesting that the assimilatory glucan is synthesized outside the plastid [104]. Surprisingly, a UDP-sugar pyrophosphorylase of the UGP family was encoded in the genome (23639) and this enzyme is predicted to be targeted to the chloroplast. The plastid localization of a potential UGP in both diatoms suggests that UDP-glucose is used for synthesis of chrysolaminaran within the CER *en route* to the vacuole. The origin of glucose-6-phosphate as substrate for a plastidal UGP remains unclear but is presumably derived from other sugar phosphates circulating in the plastid. A second candidate for UGP is one derived from a UGP/PGM fusion protein apparently localized in the cytosol (50444). This enzyme could supply UDP-glucose to a membrane-bound glucan synthase (see discussion below).

One or probably more glycosyl transferases likely synthesize the chrysolaminaran polymer, and these could be either orthologous to 1,3-β-glucan synthases in other organisms or perhaps more likely, novel enzymes due to the unique structure and function of chrysolaminaran. A single gene encoding 1,3-β-glucan synthase was identified in *P. tricornutum*. The deduced protein consists of over 2,100 amino acids and possesses 20 transmembrane domains and a signal peptide. A single 1,3-β-glucan synthase was also identified in *T. pseudonana*, although no signal peptide was detected for the predicted protein likely because the N-terminus was incompletely defined. The two diatom sequences were most similar to each other and showed similarity to callose synthase sequences from dicots such as *A. thaliana* and *Oryza sativa*. This membrane-bound enzyme likely catalyzes the addition of glucosyl residues from cytosolic UDP-glucose on one side of the membrane to the growing, non-reducing terminus of the polysaccharide chain, which protrudes from the enzyme on the opposite side of the membrane. This presumed mode of action is akin to that of plasma membrane-bound polysaccharide synthases such as the *Thalassiosira* chitin synthases [10], [111] and the cellulose synthases of terrestrial plants and multicellular algae [112], [113]. Extracellular callose has been reported in diatoms and was suggested to serve as a permeable seal in the girdle regions during cell division [103], so it is feasible that the identified 1,3-β-glucan synthase is a plasma-membrane-bound callose synthase. The relatively high EST support for this gene under a variety of growth limiting conditions, however, argues for a more active role not limited to cell division. Localization of the 1,3-β-glucan synthase to either the vacuole or the CER would help to determine where UDP-glucose is accessed from–either the cytosol or the CER. If UDP-glucose is accessed from the vacuole, this would support the hypothesis that chrysolaminaran metabolism evolved by relocating to the vacuole enzymes involved in the synthesis and processing of extracellular polysaccharides.

Three additional gene models were identified in *P. tricornutum* (48300, 56509, 50238) and in *T. pseudonana* (3105, 4956, 9237) that encode proteins with moderate similarity (up to 28%) to fungal Skn1 and Kre6, enzymes required for synthesis of fungal wall 1,6-β-glucans [114]. The diatom proteins all contain N-terminal signal peptides and single C-terminal transmembrane helices, suggesting they are also associated with the suite of enzymes that process β-glucans. Although the precise function of the fungal enzymes is unclear, they resemble family-16 glycosyl hydrolases and have been interpreted as potential glycosylases and/or transglycosylases [115]. Branching in terrestrial plant starches and mammalian and fungal glycogen is achieved by specific enzymes that hydrolyze internal α-1,4-glycosidic bonds and transfer the released reducing ends to C-6 hydroxyls of the acceptor polysaccharide chain [108], [109]. If analogous processes occur in diatoms, the putative diatom glucosylase/transglucosylases could act as chrysolaminaran branching/debranching enzymes.

### Inositol and Propanoate pathways

Many different cyclitols occur in plants with the most widespread and extensively studied being myo-inositol [116], [117]. Myo-inositol becomes incorporated into several crucial cellular compounds including those involved in signal transduction (phosphatidylinositol [PI], phosphatidylinositol-4,5-bisphosphate [PIPs]), hormone regulation (indole acetic acid [IAA] conjugates), membrane tethering (glycerophosphoinositide [GPI] anchors), stress tolerance (ononitol, pinitol), oligosaccharide synthesis (galactinol), and phosphorus storage (inositol hexakisphosphate [IP6]). Its primary breakdown product, D-glucuronic acid, is utilized for synthesis of various cell wall pectic non-cellulosic compounds, expanding the list of processes impacted by inositol synthesis and metabolism. In both *P. tricornutum* and *T. pseudonana* genes encoding enzymes predicted to be involved in inositol metabolism are well-represented, in particular the methylmalonate-semialdehyde dehydrogenase (acylating) (MMSDH), myo-inositol 2 dehydrogenase (InDH), and triosephosphate isomerase (TIM).

*De novo* synthesis of inositol has been studied mainly in yeast and proceeds from glucose 6-phosphate through inositol 1-phosphate in two steps catalyzed by inositol phosphate synthase (INPS) and inositol monophosphatase (IMP). Genes encoding these two enzymes are present in the genomes of both *P. tricornutum* and *T. pseudonana*. Myo-inositol can be interconverted to scyllo-inosose by the enzyme myo-inositol dehydrogenase (InDH; EC 1.1.1.18). Stein et al. [118] confirmed the presence of InDH in the red alga *Galdieria sulphuraria* and a further study by Gross and Meyer [119] examined the distribution of InDH in algae by confirming its presence through enzyme assays. On the basis of InDH activity they assigned the different algae tested into two distinct groups: one composed of red algae and Glaucocystophyta and the other composed of heterokontophytes and haptophytes. They also proposed an inositol/inosose shuttle across the mitochondrial membrane as an alternative to the mitochondrial NADH dehydrogenase present in higher plants and green algae. The mitochondria of red algae seem to be capable of using myo-inositol for respiration, which was hypothesized to exemplify the divergence of basic metabolism during algal evolution. In plants, neither synthesis nor degradation involves InDH and there was no InDH activity present in the green algae tested by Gross and Meyer [119]. Interestingly, neither synthesis nor catabolism involves scyllo-inosose as a reaction product in the algae studied, whereas in mammals [120] it seems to be involved in the synthesis of scyllo-inositol.

The sequences predicted to encode InDH from both *P. tricornutum* and *T. pseudonana* were compared on the basis of their predicted amino acid sequences to other InDH sequences including those from the red algae *C. merolae* and *G. sulphuraria*. The predicted protein sequences from *P. tricornutum* (51869) and *T. pseudonana* (8703) formed their own clade separate from the other sequences analysed. This would seem to provide further evidence for the theory of Gross and Meyer [119] for a divergence in algal metabolism based on InDH, but the other *P. tricornutum* sequence (34720) was found within the clade formed by the red algal sequences.

MMSDH is an enzyme involved in valine catabolism rather than inositol metabolism. Here it catalyzes the irreversible NAD+- and CoA-dependent oxidative decarboxylation of methylmalonate semialdehyde to propionyl-CoA. It has been suggested that a *Bacillus* version of the protein is located in an operon and/or involved in myo-inositol catabolism, converting malonic semialdehyde to acetyl CoA and CO_2_ [121]. Without further investigation its role in inositol metabolism in both *P. tricornutum* and *T. pseudonana* is unclear.

### There are elementary differences between green algae and diatoms

Due to their evolutionary history diatoms naturally display a completely different host cell/mitochondria/plastid relationship compared to green algae and land plants. There are clear differences between diatoms and the green algae and higher plants in the structure of thylakoids, plastid envelope membranes, the mode of carbohydrate storage and the photosynthetic properties including photoprotection (for a detailed comparison see [8], [122]). The genome of the unicellular green alga *Chlamydomonas reinhardtii*, also representing a single-celled alga but originating from a primary endocytobiosis event, has recently been sequenced [123] and perhaps not unexpectedly, most *Chlamydomonas* proteins with a plastidic function display similarity to diatom sequences. However, among the 153 diatom sequences we have analysed (table S1) only 3 of them showed the highest similarity to a *Chlamydomonas* protein whereas 23 displayed the greatest similarity to a higher plant sequence. Although the general pathways of carbohydrates are similar between diatoms and *Chlamydomonas*, several peculiar differences were identified that may have resulted from intracellular translocation of enzymes and/or pathways. Two distinctive features stand out: The mode of CO_2_ concentration in diatoms is still largely unclear, as well as the post-translational regulation of photosynthetic products.

## Materials and Methods

### Sequence analysis

We screened sequences from the current JGI (http://www.jgi.doe.gov/) diatom genome sequencing projects for the diatoms *Thalassiosira pseudonana* v3.0 (http://genome.jgi-psf.org/Thaps3/Thaps3.home.html) [10] and *Phaeodactylum tricornutum* v2.0 (http://genome.jgi-psf.org/Phatr2/Phatr2.home.html) using the BLAST algorithm [124]. Comparison with the genome sequences of the red algae *Cyanidioschyzon merolae* (http://merolae.biol.s.u-tokyo.ac.jp/) [125] and *Galdieria sulphuraria* (http://genomics.msu.edu/galdieria) [126], [127] as well as with other publicly available algal sequences helped to delimit gene modeling.

Signal peptides of endoplasmic reticulum (ER) proteins were identified using the program SignalP (http://www.cbs.dtu.dk/services/SignalP/) [128]. In addition ER proteins often possess a C-terminal retention signal. The presence of such a signal (KDEL, DDEL or DEEL) was checked manually.

Plastid proteins of diatoms possess bipartite targeting signals consisting of a signal peptide and a transit peptide-like domain with a conserved “ASAFAP”-motif at the signal peptide cleavage site [16], [17]. We screened for signal peptides using SignalP. For cleavage site predictions the results of SignalP's Neuronal networks (NN) [129] or Hidden Markov Models (HMM) [130] were used. For prediction of chloroplast transit peptide-like domains, the program ChloroP (http://www.cbs.dtu.dk/services/ChloroP/) [131] was used. The transit peptide-like domains of bipartite plastid targeting sequences often attain poor prediction scores, so we used the NCBI (http://www.ncbi.nlm.nih.gov/) Conserved Domain Search (http://www.ncbi.nlm.nih.gov/Structure/cdd/wrpsb.cgi) [132] to identify N-terminal extensions from the conserved regions of the respective protein. If a distance of at least 10 amino acids between the predicted cleavage site of the signal peptide and the region of high homology to respective proteins of other organisms was found, also a weakly predicted transit peptide-like domain was accepted. In some cases transit peptide-like domains of plastid proteins are also recognised as mitochondrial transit peptides by the program TargetP (http://www.cbs.dtu.dk/services/TargetP/) [133]. Recent mutational analysis of plastid targeting presequences revealed that only the aromatic amino acids phenylalanine, tryptophan, tyrosine and the bulky amino acid leucine at the +1 position of the predicted signal peptidase cleavage site allow plastid import [17]. Proteins which (i) possess a signal peptide but no ER retention signal (ii) possess a N-terminal extension longer than the signal peptide with some transit peptide-like features (iii) contain F, W, Y or L at the signal peptide cleavage site, were considered to be plastid targeted.

Mitochondrial transit peptides were identified using the program TargetP [133]. Putative enzymes without recognizable targeting sequences were considered cytosolic although the possibility cannot be excluded that they might be targeted to further compartments. For a detailed description of protein localization prediction see also [134].

## Supporting Information

Table S1 Genes involved in carbohydrate pathways in the diatom *Phaedactylum tricornutum* as assessed from the genome publicly available at http://genome.jgi-psf.org/Phatr2/Phatr2.home.html. For every identified gene the following information is given: enzyme name; common abbreviation; designated pathway; Protein ID; GenBank accession number (if available); number of isogenes identified; genomic coordinates; best BLAST hit: gene, organism, % identity, GenBank accession number; targeting predictions: mTP: mitochondrial targeting peptide score, SP: signal peptide score, other: probability for other localization, Loc: Prediction of localization, based on the scores of TargetP, RC: reliability class, 1 = strong, 5 = poor prediction, TPlen: length of transit peptide, regions of proposed signal peptide cleavage site; assigned localization: overall targeting prediction; respective data for homologous *T. pseudonana* genes if identified. Targeting predictions were performed by TargetP (http://www.cbs.dtu.dk/services/TargetP/) [133] and SignalP's (http://www.cbs.dtu.dk/services/SignalP/) Neuronal networks (NN) [129] or Hidden Markov Models (HMM) [130]. For a detailed description of protein localization prediction see also [134]. The Protein IDs and genomic coordinates are directly linked to the genomic database.
(0.31 MB XLS)
